# Opioids and fibromyalgia: frequency of use and factors associated with increased consumption in patients remitted to a tertiary care center

**DOI:** 10.1186/s12891-024-07263-x

**Published:** 2024-02-09

**Authors:** Javier Rivera, Juan Molina-Collada, Julia Martínez-Barrio, Belén Serrano-Benavente, Isabel Castrejón, Miguel A. Vallejo, Jose María Álvaro-Gracia

**Affiliations:** 1 Rheumatology Department, Hospital General Universitario Gregorio Marañón, Instituto de Investigación Sanitaria Gregorio Marañón (IiSGM), Calle Dr. Esquerdo, 46, Madrid, Spain; 2 Universidad Complutense de Madrid, Madrid, Spain; 3 Department of Clinical Psychology, Universidad Nacional de Educación a Distancia (UNED), Madrid, Spain

**Keywords:** Fibromyalgia, Treatment, Drug therapy, Opioids, Rheumatic diseases

## Abstract

**Background:**

Opioids are not recommended for fibromyalgia.

**Objective:**

To investigate the frequency of opioid use in a large cohort of fibromyalgia patients and to identify factors associated with opioid consumption.

**Methods:**

A retrospective, observational study of a large fibromyalgia cohort in a tertiary care center. We assessed fibromyalgia severity, functional capacity, anxiety, depression, drugs consumption and the patient’s impression of change. We compared strong opioid consumers (SOC) and non-SOC. Inferential statistical and logistic regression analysis were used to identify factors associated with opioid consumption, and ANOVA for repeated measurements.

**Results:**

We found a prevalence of 9.2% of SOC (100 patients) among 1087 patients in the cohort. During the last four years there was a significant increase on the incidence of SOC up to 12.8% (*p* = 0.004). There were no differences in demographic variables between SOC and non-SOC. Clinical variables were significantly more severe in SOC, and they consumed more non-opioid drugs (*p* < 0.0001). Opioid consumption was independently associated with other non-opioid drugs (Odds ratio 1.25, CI: 1.13–1.38), but not with the fibromyalgia severity. At three months, 62% of the patients had opioid withdrawal. There were no statistical differences in the fibromyalgia severity at the initial evaluation, or the patient’s impression of change compared with those patients who continued opioids. Coping strategies were better in those patients who withdrew opioids (*p* = 0.044).

**Conclusions:**

We observed an increase in opioid prescriptions during the last four years. Opioid consumption was associated with concomitant use of non-opioid drugs, but it was not associated with fibromyalgia severity.

## Introduction

The recent widespread availability of oral opioids for several chronic pain disorders has led to their use in the management of fibromyalgia (FM). After an initial peak in USA twenty years ago, followed by an increase in other countries such as Australia and Canada [1], the opioid crisis has caused an alarming increase of deaths requiring a drastic intervention of the Health Authorities in that country [2]. Although in Spain and other European countries the increase in opioid prescriptions has not been so alarming [3], Health Authorities have detected an increase in prescription, which has risen some concerns [4].

Risks associated with the use of opioids are well known, and include over dosage, abuse, suicides and deaths, among the most important adverse events [5].

The frequency of opioid treatment in patients with chronic musculoskeletal pain is around 20%, depending on the country, the type of the study, the disease itself and whether strong or weak opioids are considered at the same time [6].

However, the efficacy of the opioid treatment in patients with chronic pain is controversial with small benefits in some cases, or the absence of a good evidence for its use in others [7]. Although the efficacy of opioids in the treatment of FM is questionable [1], prescriptions have increased over time from studies in the 1990s with a prevalence of 14% [8] to most recent studies with a prevalence of 24% [6]. In these patients, an impaired response to opioid treatment has been described due to a decreased opioid binding potential because the endogenous opioid system is already activated [9]. Furthermore, it is known that opioids affect the activation of spinal glia that enhances pain transmission [10]. As FM is a central sensitization syndrome with a certain degree of neuroinflammation and glial activation [11], the use of opioids in these patients could cause a contrary effect, increasing the level of pain.

In addition, some therapeutical guidelines do not recommend its use in patients with FM [12–16], and the pattern of opioids use and the factors associated with it are not well understood.

The main objective of this study is to describe the frequency of patients with FM who are treated with opioids and to identify factors associated to an increased opioid consumption in a well-defined cohort of patients with FM treated in a specialized care centre during the last decade.

## Methods

### Study design

This is a retrospective observational study of a cohort of FM patients (the Combined Index of Severity in FM (ICAF) cohort) conducted in a tertiary care university teaching hospital in Spain.

Patients at first visit (baseline) were divided in two groups: strong opioid consumers (SOC patients) and not consumers (Non-SOC patients), if they were taking strong opioids or not when they were remitted to our unit (first visit). In this visit main demographic data, ICAF questionnaire [17], FM criteria [18] and information about the drugs taken by the patient at the moment of inclusion in the cohort were collected. We compared clinical variables and drug treatment between both groups at this visit.

A new treatment was later prescribed by the rheumatologists of our unit in conditions of routine care following EULAR recommendations [12], and with a special emphasis on opioids withdrawal.

At the second visit (3 months follow-up visit), patients completed the ICAF questionnaire, drug treatment was recorded again and patients also completed the Patient Global Impression of Change (PGIC) [19] questionnaire to evaluate their overall health status. At this visit, we analyzed clinical variables in the group of SOC patients, comparing those who kept on opioid therapy with those who withdrew opioids.

We also evaluated the possible increase in the prevalence of SOC patients during the duration of the cohort, analyzing the frequency of SOC patients in three different periods of four years each between 2010 and 2022.

### Patients

Patients were derived at our FM-specialized unit from the general practitioner or from other specialists for the treatment and control of the disease. All patients came to our unit with the treatment previously prescribed by the remitting doctor.

The ICAF cohort started in 2010 and the objectives of this cohort were to characterize the clinical course through the ICAF questionnaire [17] and the use of drug treatments. Patients in this cohort were evaluated at baseline, three months later and yearly afterwards to complete a five year follow-up. Inclusion criteria for entry in this cohort were: being older than 18 years and fulfilling the 2010 American College of Rheumatology (ACR) FM criteria [18]. For this study, we excluded all those patients with another severe disease associated with FM when they were remitted to our unit.

We considered as SOC all those patients taking any of the approved opioids by the Agencia Española del Medicamento y Productos Sanitarios (Spanish Agency for Therapeutical Drugs and Sanitary Products), including codeine, hydrocodone, oxycodone, oxymorphone, meperidine, fentanyl, buprenorphine, hydromorphone, tapentadol and morphine. Tramadol is considered as a weak opioid with a 0.1 mg morphine equivalent dose quite different from the morphine equivalent dose of, for example, fentanyl, which is 2.4 mg. We also did not include tramadol in our study because in Spain it is frequently used as a pain killer with doses as low as 37.5 mg/day.

Although ibuprophen is a non-steroidal antinflammatory drug, we included it as an analgesic drug because it is usually taken at a sub-therapeutical dosages.

### Questionnaires

ICAF [17, 20] is a specific quality-of-life questionnaire for FM patients, with a total score of the severity of the disease along with four other factors. The physical factor measures the functional ability from the point of view of physical capacity to perform daily activities; the emotional factor measures the severity of clinical manifestations such as anxiety and depression; and two factors of active and passive coping measure the attitude of the patient in coping with the disease. All scores are expressed on a T-scale with an average of 50 and a standard deviation of 10. A high score indicates greater severity, except for the active coping factor that indicates a better coping.

The importance of each factor over the total score is different, so the emotional and physical factors are the ones with the highest weight while each coping factor only contributes a small part of the total score severity of the disease [20]. This questionnaire has been previously validated in our population [17].

The ACR criteria [18, 21] are constructed with two variables: the widespread pain index (WPI) (range, 0–19) and the symptomatic severity scale (SSS) (range, 0–12). The sum of both conforms the polysymptomatic distress scale (PSD) (range, 0–31) [21] which measures the global severity of FM.

The SSS itself is the sum the 0–3 severity scores of the 3 principal symptoms: fatigue, waking unrefreshed, and cognitive symptoms (total score, 0–9), plus the sum of the presence of headaches, pain or cramps in lower abdomen and depression (total score, 0–3).

The PGIC [19] is a specific questionnaire to evaluate overall improvement after an intervention. It includes a Likert scale from 1 to 7 (1 = much better, 2 = better, 3 = a little better, 4 = equal, 5 = a little worse, 6 = worse or 7 = much worse) to assess how the patient is feeling after an intervention. This questionnaire has been validated in patients with FM [22] to assess the perception of overall improvement experienced after a therapeutic intervention and is widely used in studies on chronic pain and FM.

### Statistical analysis

For statistical analysis, t-test was used to compare continuous variables and chi-square test for categorical variables.

To identify factors associated with opioid consumption, a logistic regression analysis with a backward selection was performed introducing in the model all the clinical and demographic variables that showed statistical significance in the univariate analysis. We used a *p* < 0.05 for including variables in the multivariate model.

To analyze the trend of the frequency of the numbers of SOC patients throughout the total duration of the ICAF cohort, we used a a regression model with a trend time.

To analyze the evolution between the ICAF-pre and the ICAF-post, an ANOVA for repeated measurements was used.

## Results

Between December 2010 to July 2022, 1342 FM patients were included in the ICAF cohort. Of them, 1087 patients fulfilled the inclusion criteria for this study and were included for analysis. Of the 255 FM patients excluded from the study, 242 patients were due to the presence of another severe comorbidity such as inflammatory arthritis, connective disease, or cancer. In 13 patients, data about drug treatment were missed and were also excluded from the study.

**Fig. 1:**
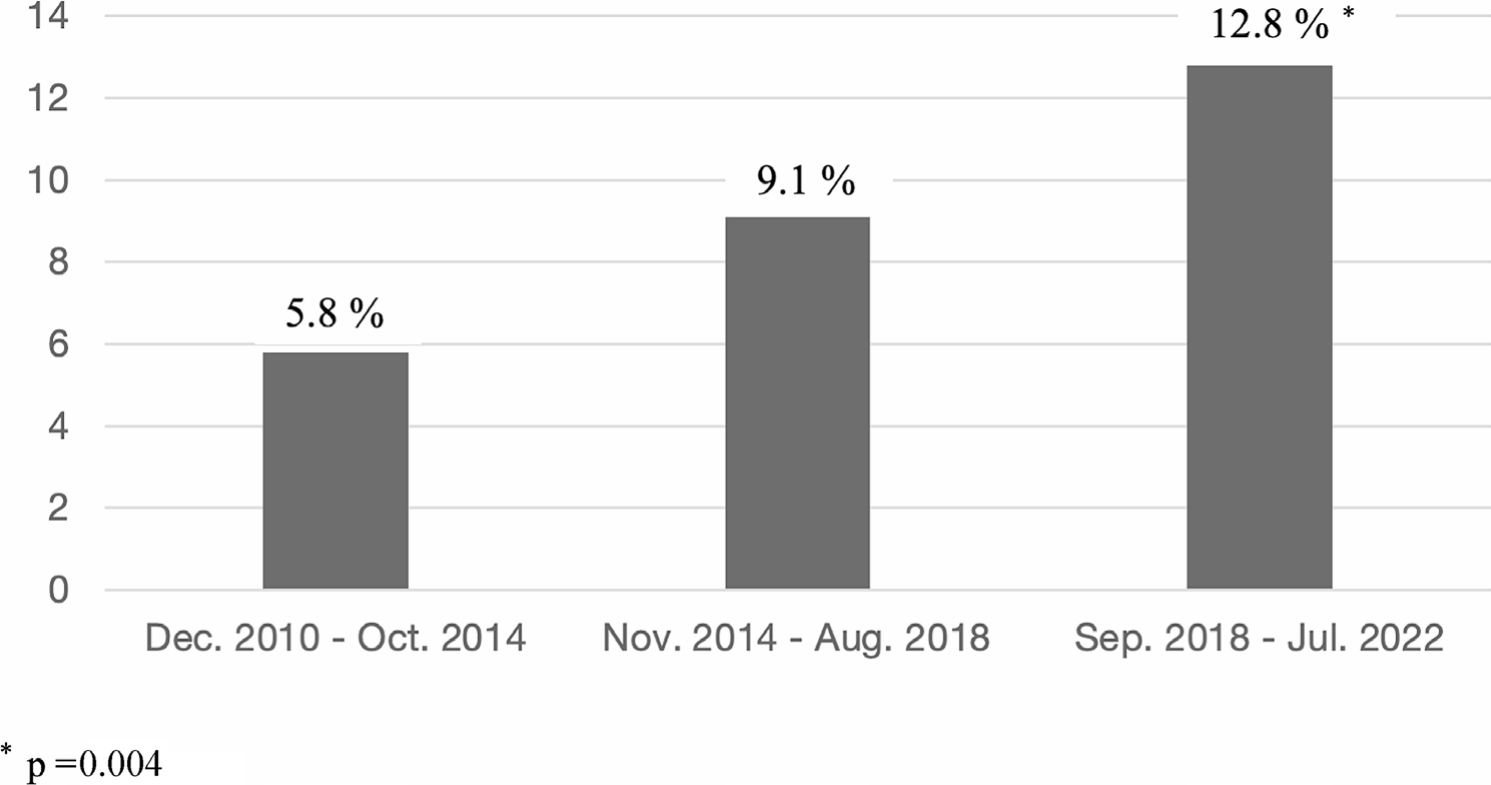
Increasing percentages of SOC patients along the three different periods

There was a progressive and significant increase in the frequency of SOC patients during the three different periods in which we divided the total time of the cohort, as can be seen in Table 1; Fig. 1.

**Table 1:** FM patients included in the ICAF cohort, and SOC patients in each period

	Dec. 2010 – Oct. 2014	Nov. 2014 – Aug. 2018	Sep. 2018 – Jul. 2022	Total	p*
FM patients included in the ICAF cohort	327 (30.1%)	439 (40.4%)	321 (29.5%)	1087 (100%)	
SOC patients	19 (5.8%)	40 (9.1%)	41 (12.8%)	100 (9.2%)	**0.004**

FM = fibromyalgia. SOC = strong opioid consumer

P* compares the difference in trend between the periods of time

There were 100 (9.2%) SOC patients in the ICAF cohort with a total of 110 opioids prescriptions distributed as follows: tapentadol, 32 (29.1%); oxycodone, 30 (27.3%); fentanyl, 22 (20.0%); codeine, 16 (14.5%); morphine, 6 (5.4%); buprenorphine, 3 (2.7%), and hydromorphone 1 (0.9%). One patient was treated with three different types of opioids and eight patients with two opioids at the same time, usually as a rescue therapy for pain.

Only 6.3% of patients were males in the ICAF cohort, with no statistical differences between both groups (Table 2).

**Table 2:** Comparison between the SOC group and the Non-SOC group at the first visit. Total number, percentages, sex and mean age of the patients

	SOC group 100 patients		Non-SOC group 987 patients		
	Number (%)	Mean age (sd)	Number (%)	Mean age (sd)	p*
Female	90 (90.0%)	49.0 (8.3)	928 (91.2%)	48.35 (9.4)	0.256
Male	10 (10.0%)	52.9 (4.1)	59 (8.8%)	44.71 (8.4)	**0.004**

*p* * compares mean age of the SOC group vs. Non-SOC group

SOC = strong opioid consumer. sd = standard deviation

Among the SOC group, there was a higher percentage of males, but it did not reach statistical significance compared with the percentage of male patients in the Non-SOC group. Mean age of males in the SOC group was higher than females but also did not reach statistical significance (Table 2).

The mean age of the SOC group males was higher and statistically significant, compared with the Non-SOC group males (Table 2).

There was no statistical difference in mean age between the SOC and non-SOC groups (49.4 (8.1) vs. 48.1(9.4); *p* = 0.256).

All severity scores for FM, including ACR criteria and ICAF scores, were significantly worse in SOC patients compared with non-SOC patients (Table 3).

**Table 3:** Main clinical variables in the SOC group compared with the non-SOC group at the first visit

	SOC group Mean (sd)	Non-SOC group Mean (sd)	*p*
**Number of patients**	100	987	
**ACR criteria**			
Widespread pain index (0–19)	14.9 (3.2)	14.0 (3.3)	**0.005**
Three principal symptoms (0–9)	7.7 (1.4)	7.3 (1.5)	**0.014**
Severity of somatic symptoms (0–3)	2.6 (0.7)	2.4 (0.8)	**0.022**
Polysymptomatic distress scale (0–31)	25.16 (4.5)	23.7 (4.4)	**0.003**
**ICAF**			
Physical factor	57.6 (8.8)	53.7 (8.9)	**< 0.0001**
Emotional factor	57.6 (11.0)	52.7 (10.1)	**< 0.0001**
Active coping	49.0 (10.4)	51.3 (10.0)	**< 0.0001**
Passive coping	56.4 (9.7)	52.0 (10.8)	**< 0.0001**
Total score	58.2 (10.1)	52.8 (9.9)	**< 0.0001**

ACR = American College of Rheumatology. ICAF = Combined Index of Severity in Fibromyalgia questionnaire. SOC = strong opioid consumer. sd = standard deviation

In parallel with increased opioids consumption, SOC patients also showed an increased consumption of other medication to control FM symptoms in comparison with Non-SOC patients (*p* < 0.0001) (Table 4).

**Table 4:** Percentages of patients treated with additional drugs, and total number of drugs consumed by all groups of patients at the first visit

	ICAF cohort	SOC patients	Non-SOC patients	*p* ^*^
Analgesics	75.3	75.0	75.3	0.999
NSAIDs	46.6	41.0	47.1	0.347
Benzodiazepines	56.2	73.0	54.5	**< 0.0001**
Antidepressants	63.7	86.0	61.5	**< 0.0001**
Anticonvulsants	26.6	57.0	23.5	**< 0.0001**
Z-drugs	5.4	9.0	5.1	0.105
Antipsychotics	2.8	6.0	2.4	**0.038**
Antihistamines	5.2	10.0	4.8	**0.025**
Other CNS drugs	9.9	16.0	9.3	**0.033**
Total number of drugs^**^ (mean (sd))	3.7 (2.1)	4.9 (2.1)	3.6 (2.1)	**< 0.0001**

p^*^ Compares SOC vs. non-SOC groups. ^**^ Strong opioids are not included. SOC = strong opioid consumer sd = standard deviation. NSAIDs = non-steroidal anti-inflammatory drugs. CNS = central nervous system. ns = not significant

Almost all types of drugs were significantly more frequent in the SOC group except for analgesics, NSAIDs, z-drugs or other central nervous system drugs (Table 4).

There was no statistical difference between females and males in the total number of drugs consumed, a total of 3.8 (2.2) in females *versus* 3.6 (2.5) in males.

In a logistic regression model, the only variable associated with opioid consumption was the total number of non-opioid drugs consumed by the patient (Odds ratio 1.25. CI (1.13–1.38)). The univariate analysis of the variables introduced in the logistic regression model is showed in Table 5.

**Table 5:** Univariate analysis of the variables introduced in the regression logistic model

	Score	df	*p*
Widespread pain index	6.694	1	0.010
Severity score of the three principal symptoms	4.982	1	0.026
Severity of somatic symptoms	5.457	1	0.019
ICAF Total score	23.746	1	< 0.001
ICAF Physical factor	16.912	1	< 0.001
ICAF Emotional factor	19.488	1	< 0.001
ICAF Passive coping factor	12.178	1	< 0.001
Sum of the drugs (opioids are not included)	35.667	1	< 0.001

df = degrees of freedom. sd = standard deviation

Of the 100 SOC patients, we obtained additional data with a 3-months follow-up for 69 over 100 patients in the SOC group. A total of 26/69 (38%) patients kept on opioid consumption, whereas 43/68 (62%) had withdrawal opioids (Table 6).

**Table 6:** Patients on the SOC group: differences between patients who kept on opioid therapy and those who withdrew opioids at the second visit

Second visit at 3 months	SOC patients		
	kept on opioids	withdrew opioids	*p*
**Number of patients**	26	43	
**ICAF (mean (sd)**			
Physical factor	54.7 (11.0)	53.7 (13.8)	0.770
Emotional factor	57.1 (12.1)	53.7 (12.0)	0.275
Active coping	48.8 (11.0)	54.3 (11.0)	0.051
Passive coping	55.5 (10.0)	54.8 (10.7)	0.784
Total score	56.9 (13.1)	52.7 (11.7)	0.180
**PGIC (percentages)**			0.898
Better	31%	30%	
The same	23%	28%	
Worse	46%	42%	

SOC = strong opioid consumer. sd = standard deviation. ICAF = Combined Index of Severity in Fibromyalgia. PGIC = patient global impression of change. ns = not significant

The comparison between these groups of patients at the second visit showed no statistical differences in all ICAF scores. The PGIC did not show any statistical difference between both groups as well. In relation with drugs consumption, patients who kept on opioid still consumed more drugs different from opioids than patients who withdrew opioids (5.5 (2.4) *versus* 4.2 (1.6); *p* < 0.001).

We did not find differences on clinical variables at baseline between patients stopping or maintaining opioids therapy, except for the active coping factor, which was better in patients stopping opioids at the 3 months follow-up (51.7 (9.9) *versus* 46.7 (9.5), *p* = 0.044).

## Discussion

In this work, we found a progressive increase in the percentage of SOC patients along the twelve-year duration of the study, especially during the last four years that reached up to 12.8% of the patients. This percentage of FM patients taking opioids in our cohort is considerably lower than in other series, as in USA reaching 21% [23], 23% in Canada [24] or a median of 24% found in a recent systematic review [6]. We believe that the lower percentage of SOC patients found in our study is not due to the fact that we did not include tramadol in the analysis with a possible underestimation of real percentages because another study about the global use of opioids in our country found a similar number of SOC patients [4]. Moreover, there are some studies with a high prevalence of SOC patients among FM population in which tramadol was also excluded from the analysis [24].

In the USA the number of opioid prescriptions has dropped since 2012, once the high percentages of serious adverse events associated with opioid consumption in the general population were discovered. Furthermore, in our country we found a progressive increase of strong opioid prescriptions in FM patients throughout the last four years, in spite of the fact that most of the principal therapeutical guidelines in FM do not recommend its use for treating FM patients [12–16].

A similar trend has been observed in other countries, such as Australia and Canada, with an increasing use of opioids, but not in Germany [1].

The reasons for opioid prescription in FM patients may be multiple. The severity of pain may induce to prescribe opioids when there are unrealistic expectations about pain control by opioids [25]. On the other hand, patients with FM consider opioids as the best symptom reliever, as pointed out in an Internet survey [26], and may add pressure to the prescription by the attending physicians.

As for the characteristics of the SOC patients associated with opioid consumption, we did not find differences in demographic variables compared with the non-SOC patients, but all the clinical variables on FM severity were worse in SOC patients. Moreover, the number of concomitant drugs for treating FM in our SOC patients were also higher as well, specifically benzodiazepines, antidepressants, anticonvulsants, antipsychotics and antihistamines. Similar findings about a higher severity of symptoms in opioids consumers compared with non-consumers FM patients have been previously reported [27, 28].

We found that the only variable associated with opioid consumption was the total number of other drugs used for treating FM, but there were no associations with other demographical or clinical variables of FM severity. Other studies have shown association between opioid consumption and negative psychosocial factors, such as unemployment, drug abuse or mental health [24]. In other series, the association was with administrative or prescription policies of different geographical areas [23], or, as in our study, there was no association with clinical variables of FM severity [27]. All these findings suggest that opioid consumption mostly depends on external factors rather than on the severity of the FM itself.

A possible explanation for a greater severity in clinical variables in the SOC patients in our cohort is the numerous adverse effects of opioids and other nervous system acting drugs used for treating FM in these patients. In this sense, some of the most common adverse side effects of opioids, such as opioid-induced hyperalgesia, cognitive impairment, sleep alterations, fatigue or constipation are symptoms of FM as well, and the opioid treatment may produce an increase of these symptoms with a global impairment in the patients and the severity of the disease.

Opioid treatment has not shown a good level of evidence for efficacy in FM, except for the weak opioid tramadol in two randomized clinical trials that showed some improvement on pain [29, 30]. Furthermore, about half of the patients with chronic pain continue maintaining high levels of pain in spite of the opioid treatment [31]. Other studies have also found that opioid treatment does not improve the general situation of the FM patients [28, 32].

As for the efficacy of other treatments in FM patients, a multidisciplinary treatment program [33] did not show efficacy in patients taking opioids, but it was effective in those patients who did not consume opioids. Similar results were also found with a cognitive behavior therapy program [34].

In patients with a 3-month follow-up in our cohort we did not observe any clinically significant difference between the group of patients maintaining or stopping opioids, which suggests that opioid treatment did not add any advantage to the treatment of our FM patients. However, coping strategies at baseline were better in patients who were able to stop opioids therapy after three months.

In a recent pain rehabilitation program for opioids tapering carried out in a group of FM patients, the authors found an improvement of pain-related measures and health perception after opioids cessation [35]. This is not an isolated finding, and some other studies found similar results when opioid treatment is stopped in these patients [36, 37].

It is known that deaths associated with opioid consumption are very high [38] and multiple causes such as respiratory failure or suicide are related to this. In the case of FM, the mortality associated with this disease is also increased compared with the general population [39] and the use of opioid therapy could be related to this high mortality.

In the treatment of a chronic musculoskeletal pain and FM the use of antidepressants, benzodiazepines or gabapentinoids are very common, and all these drugs may produce a respiratory depression contributing to increasing the number of deaths in combination with opioids use [40]. Concomitant treatment with benzodiazepines and opioids multiplies by fifteen the risk of death comparing with patients who do not consume any of these drugs [41], and there is an increase of 50% of deaths in patients treated with opioids and gabapentin [42].

Although at present, there is not good scientific evidence about the efficacy of opioid therapy for the treatment of pain in patients with FM, all these arguments strongly advise against the use of this treatment in these patients. All the physicians involved in the treatment of FM patients should be aware of the severe risks associated with the use of opioids in these patients.

The strength of our work lies in the large cohort of FM patients followed during a long period of time with periodic evaluations.

The main limitation of this study is that it has been performed only in one center specialized in FM management and this does not allow to generalize the results to other centers. These results need to be evaluated in other cohorts of FM patients.

Although there may be a risk for overestimation of the frequency of SOC among patients with FM due to the fact that this study has been performed in patients remitted to a specialized FM center, the increased frequency of SOC during the duration of this cohort probably reflects a real tendency of opioid treatment among patients with FM. Another limitation of our work is the retrospective design with all the corresponding limitations of this type of studies.

In conclusion, the most important finding of our study is the progressive increase in opioids prescription among FM patients remitted to a tertiary care center during the last years. Opioid treatment is not related to the severity of FM but to the number of other drugs prescribed for treating these patients. Some results of this study suggest that opioid treatment did not add any advantage to the treatment of FM patients.

## Data Availability

The datasets used and/or analysed during the current study are available from the corresponding author on reasonable request.

## References

[1] Häuser W, Schug S, Furlan AD (2017). The opioid epidemic and national guidelines for opioid therapy for chronic noncancer pain: a perspective from different continents. PAIN Rep.

[2] Volkow ND, Collins FS (2017). The role of Science in addressing the Opioid Crisis. N Engl J Med.

[3] Fuster D, Muga R (2018). La crisis de Los opioides. Medicina Clínica.

[4] Agencia Española de Medicamentos y productos Sanitarios. Utilización de medicamentos opioides en España durante el periodo 2008–2015. 2017. Available from: https://www.aemps.gob.es/medicamentosUsoHumano/observatorio/docs/opioides-2008-2015.pdf.

[5] Chou R, Turner JA, Devine EB, Hansen RN, Sullivan SD, Blazina I (2015). The effectiveness and risks of Long-Term Opioid Therapy for Chronic Pain: a Systematic Review for a National Institutes of Health Pathways to Prevention Workshop. Ann Intern Med.

[6] De Sola H, Dueñas M, Salazar A, Ortega-Jiménez P, Failde I (2020). Prevalence of therapeutic use of Opioids in Chronic Non-cancer Pain patients and Associated factors: a systematic review and Meta-analysis. Front Pharmacol.

[7] Trouvin AP, Berenbaum F, Perrot S (2019). The opioid epidemic: helping rheumatologists prevent a crisis. RMD Open.

[8] Wolfe F (1997). A prospective, longitudinal, multicenter study of service utilization and costs in fibromyalgia. Arthritis Rheum.

[9] Harris RE, Clauw DJ, Scott DJ, McLean SA, Gracely RH, Zubieta JK (2007). Decreased central -opioid receptor availability in Fibromyalgia. J Neurosci.

[10] Watkins L, Watkins LR, Hutchinson MR, Johnston IN, Maier SF. Glia: novel counter-regulators of opioid analgesia. Trends Neurosci. 2005;28(12):661-9. Trends Neurosci. 2005;28(12):661–9. 10.1016/j.tins.2005.10.001 16246435

[11] Albrecht DS, Forsberg A, Sandström A, Bergan C, Kadetoff D, Protsenko E (2019). Brain glial activation in fibromyalgia – a multi-site positron emission tomography investigation. Brain Behav Immun.

[12] Carville SF, Arendt-Nielsen S, Bliddal H, Blotman F, Branco JC, Buskila D (2008). EULAR evidence-based recommendations for the management of fibromyalgia syndrome. Ann Rheum Dis.

[13] Eich W, Häuser W, Arnold B, Jäckel W, Offenbächer M, Petzke F (2012). Das Fibromyalgiesyndrom: definition, Klassifikation, Klinische diagnose und prognose. Schmerz.

[14] Macfarlane GJ, Kronisch C, Dean LE, Atzeni F, Häuser W, Fluß E (2017). EULAR revised recommendations for the management of fibromyalgia. Ann Rheum Dis.

[15] Rivera Redondo J, Díaz del Campo P, Alegre de Miquel C, Almirall Bernabé M, Casanueva Fernández B, Castillo Ojeda C (2022). Recommendations by the Spanish Society of Rheumatology on Fibromyalgia. Part 1: diagnosis and treatment. Reumatol Clin.

[16] Ariani A, Bazzichi L, Sarzi-Puttini P, Salaffi F, Manara M, Prevete I (2021). The Italian Society for Rheumatology clinical practice guidelines for the diagnosis and management of fibromyalgia. Best practices based on current scientific evidence. Reumatismo.

[17] Vallejo MA, Rivera J, Esteve-Vives J, Rejas J, Group ICAF (2011). A confirmatory study of the Combined Index of Severity of Fibromyalgia (ICAF*): factorial structure, reliability and sensitivity to change. Health Qual Life Outcomes.

[18] Wolfe F, Clauw DJ, Fitzcharles MA, Goldenberg DL, Katz RS, Mease P (2010). The American College of Rheumatology Preliminary Diagnostic Criteria for Fibromyalgia and Measurement of Symptom Severity. Arthritis Care Res.

[19] Guy W. ECDEU Assessment Manual for Psychopharmacology. Washington DC. USA: Goverment Printing Office; 1976. Available from: https://openlibrary.org/books/OL24341821M/ECDEU_assessment_manual_for_psychopharmacology.

[20] Vallejo MA, Rivera J, Esteve-Vives J, Group ICAF (2010). Development of a self-reporting tool to obtain a combined index of severity of Fibromyalgia (ICAF*). Health Qual Life Outcomes.

[21] Wolfe F, Clauw DJ, Fitzcharles MA, Goldenberg DL, Häuser W, Katz RS (2011). Fibromyalgia Criteria and Severity scales for Clinical and Epidemiological studies: a modification of the ACR Preliminary Diagnostic Criteria for Fibromyalgia. J Rheumatol.

[22] Rampakakis E, Ste-Marie PA, Sampalis JS, Karellis A, Shir Y, Fitzcharles MA (2015). Real-life assessment of the validity of patient global impression of change in fibromyalgia. RMD Open.

[23] Painter JT, Crofford LJ, Talbert J (2013). Geographic Variation of Chronic Opioid Use in Fibromyalgia. Clin Ther.

[24] Fitzcharles MA, Ste-Marie PA, Gamsa A, Ware MA, Shir Y (2011). Opioid Use, Misuse, and abuse in patients labeled as Fibromyalgia. Am J Med.

[25] Krashin D (2013). What are we treating with chronic opioid therapy?. Curr Rheumatol Rep.

[26] Bennett RM, Jones J, Turk DC, Russell IJ, Matallana L (2007). An internet survey of 2,596 people with fibromyalgia. BMC Musculoskelet Disord.

[27] Bruce BK, Allman ME, Rivera FA, Abril A, Gehin JM, Oliphant LM (2021). Opioid use in Fibromyalgia continues despite guidelines that do not support its efficacy or risk. J Clin Rheumatol.

[28] Peng X, Robinson RL, Mease P, Kroenke K, Williams DA, Chen Y (2015). Long-term evaluation of Opioid Treatment in Fibromyalgia. Clin J Pain.

[29] Bennett R (2003). Tramadol and acetaminophen combination tablets in the treatment of fibromyalgia pain: a double-blind, randomized, placebo-controlled study. Am J Med.

[30] Biasi G (1998). Tramadol in the fibromyalgia syndrome: a controlled clinical trial versus placebo. Int J Clin Pharmacol Res.

[31] Wasserman RA, Brummett CM, Goesling J, Tsodikov A, Hassett AL. Characteristics of Chronic Pain patients who take opioids and persistently Report High Pain Intensity: Regional Anesthesia and Pain Medicine. 2014;39(1):13–7. 10.1097/AAP.0000000000000024 PMC3960717 24310048

[32] Ngian GS, Guymer EK, Littlejohn GO (2011). The use of opioids in fibromyalgia: use of opioids in fibromyalgia. Int J Rheum Dis.

[33] Hwang Jmoon, Lee B, joo, Oh TH, Park D, Kim Chyun (2019). Association between initial opioid use and response to a brief interdisciplinary treatment program in fibromyalgia. Medicine.

[34] McCrae CS, Curtis AF, Miller MB, Nair N, Rathinakumar H, Davenport M (2020). Effect of cognitive behavioural therapy on sleep and opioid medication use in adults with fibromyalgia and insomnia. J Sleep Res.

[35] Cunningham JL, Evans MM, King SM, Gehin JM, Loukianova LL (2016). Opioid tapering in Fibromyalgia patients: experience from an Interdisciplinary Pain Rehabilitation Program. Pain Med.

[36] Fishbain DA, Pulikal A (2019). Does Opioid Tapering in Chronic Pain patients result in Improved Pain or same Pain vs increased Pain at Taper Completion? A structured evidence-based systematic review. Pain Med.

[37] Hooten W (2007). Treatment outcomes after multidisciplinary pain rehabilitation with analgesic medication withdrawal for patients with fibromyalgia. Pain Med.

[38] Rudd R (2016). Increases in drug and opioid-involved overdose deaths - United States, 2010–2015. MMWR Morb Mortal Wkly Rep.

[39] Calandre E (2011). Suicide attempts and risk of suicide in patients with fibromyalgia: a survey in Spanish patients. Rheumatology (Oxford).

[40] Babalonis S, Walsh SL (2015). Risks of co-prescribing opioids and benzodiazepines. Pain Clin Updates.

[41] Pierce G (2012). Doctor and pharmacy shopping for controlled substances. Med Care.

[42] Gomes T, Juurlink DN, Antoniou T, Mamdani MM, Paterson JM, van den Brink W. Gabapentin, opioids, and the risk of opioid-related death: A population-based nested case–control study. Tsai AC, editor. PLoS Med. 2017;14(10):e1002396. 10.1371/journal.pmed.1002396 PMC5626029 28972983

